# Building carbon structures inside hollow carbon spheres

**DOI:** 10.1038/s41598-019-46992-1

**Published:** 2019-07-23

**Authors:** Prakash M. Gangatharan, Manoko S. Maubane-Nkadimeng, Neil J. Coville

**Affiliations:** 1 DST-NRF Centre of Excellence in Strong Materials and the Molecular Sciences Institute, School of Chemistry, University of the Witwatersrand, 2050 Johannesburg, South Africa; 2 Microscopy and Microanalysis Unit, University of the Witwatersrand, 2050 Johannesburg, South Africa

**Keywords:** Catalyst synthesis, Nanoparticles, Synthesis and processing

## Abstract

The synthesis and characterization of helical carbon nanofibers (CNFs) contained within a fully confined nanoreactor is described. In particular, hollow carbon spheres (od = ca. 310 nm; wall thickness ca. 20 nm) were infiltrated with Cu ions (1%) to produce CuO particles (<10 nm) and the CuO was converted to Cu particles at temperature of 300 °C. Acetylene was then used as a carbon source to grow helical CNFs within the hollow carbon spheres. The diameter and helicity of the CNFs was influenced by the Cu content within a hollow carbon sphere, the limited Cu sintering inside a sphere as well as the dimensions of the sphere. The procedures employed suggest that the philosophy of building other structures (and molecules) with any elements within confined nanoreactors is possible.

## Introduction

In 1959, Feynman delivered his often-quoted lecture in which he outlined proposals for the development of nanotechnology concepts^1^. Soon afterwards, Drexler wrote his book ‘Engines of Creation’ in which the ideas for making nanomachines was outlined^2^. This led to the debates between Smalley and Drexler (and others) on the feasibility of making and using nanomachines^3,4^. While these issues may not have been resolved, the ability to make molecules that can act as nanomachines and can ‘move’ under appropriate conditions has been accomplished. This is exemplified by the award of the 2016 Noble Prize in Chemistry, as a reward for studies in which molecular motion was achieved and harnessed^5^.

While the focus of the above issues has been on motion at the nano level, the ability to create *structures* at the nano level has gone on unabated. Control of nanostructures using catalysts, capping agents, templates etc. has led to an understanding of the methods used to grow nanostructured materials^6–8^. In this article, we wish to focus on an issue that relates to growing nanostructures in a *large* nano-confined environment. Indeed the ability to generate new structures in *small* nano*-*confined environments is well known, for example, as seen in metal and carbon growth in carbon nanotubes^9–11^ and zeolites^12^. However, these materials (metals/oxides) are formed in open-ended structures, and the confinement is not in all dimensions.

To synthesize nanostructured materials that are *fully confined* and to develop simple procedures to make nanostructures with controlled dimensionality, with in principle any element, we have used hollow carbon spheres (HCSs) as our model container. This approach has similarities to the ‘ship in the bottle’ philosophy used to make macro (and micro^13^) structures in a constrained environment. In our approach, the structures are not made prior to addition to the container, but are made inside the container. We have chosen to construct known carbon materials inside HCSs as a proof of concept to the problem of making nanostructured materials in a fully confined environment.

Routes to making HCSs are well known, and the properties of these HCS materials have been explored^14^. The porosity of the HCSs can be varied to allow different sized molecules to enter and exit the HCS^15^. This allows small building blocks to pass into the HCS, and after construction has taken place, the larger pieces made from the smaller building blocks remain entrapped within the HCSs.

This process is possible when the building blocks are metals. For example, Cu ions have been impregnated into a HCS and under appropriate conditions, the Cu ions form Cu particles that sinter^16^ and cannot escape the confines of the HCS. In contrast, the *growth of non-metal materials* inside a HCS has not been explored. In this paper, we describe procedures for using carbon molecules to build carbon structures inside a HCS.

In particular, we have chosen to grow helical carbon fibres inside a HCS using a Cu catalyst^16^. We have chosen these helical carbons for study as they can be prepared at low temperature and the helix properties are influenced by the Cu particle morphology. Indeed numerous studies have indicated that the size of the particle^17–19^ as well as the particle shape^20^ influence the growth of the carbon helix. However, control of the growth of the Cu particle on a typical support (or if unsupported) is difficult as Cu readily sinters under the reaction conditions. Use of a HCS provides one method for limiting sintering of the Cu to the Cu content found only inside the HCS. As will be described, it is thus possible to observe and study controlled helix growth inside a HCS.

Numerous implications from this study can be envisaged. (i) The ability to build structures inside a HCS that are larger than the pores of the HCS shell will allow for the construction of large molecules from smaller building blocks within the confines of the hollow sphere. For example, addition of a metal ion (M^+^) and ligand (L) into the HCS, and the correct choice of reaction conditions, will allow for construction of ML_n_^+^ species that cannot exit the HCS. In this way homogeneous catalysts and other complexes could be constructed within a HCS. (ii) This approach then leads to the ability to filter homogeneous catalysts trapped inside a HCS, a limiting constraint in the use of industrial homogeneous catalysts. (iii) Many heterogeneous catalysts disintegrate under reaction conditions but if contained within a HCS the fine particles could be trapped inside the HCS.

## Results and Discussion

**Figure 1:**
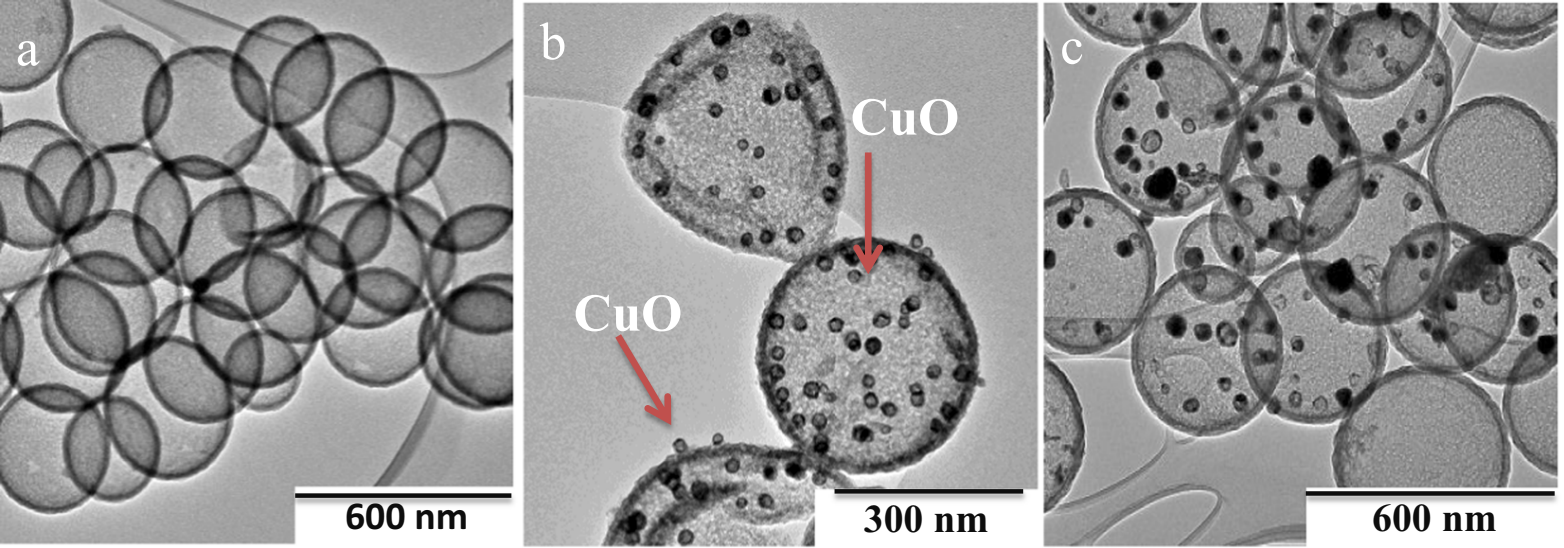
TEM image of (**a**) HCSs, (**b**) CuO@HCS (5% loading) before reduction and (**c**) Cu@HCS (5% loading) after reduction under H_2_ at 400 °C for 1 h. Note that images (**b**,**c**) are taken from different collections of HCSs, but from the same sample. The arrows in (**b**) indicate that the CuO particles are found both inside and outside the HCS.

**Figure 2:**
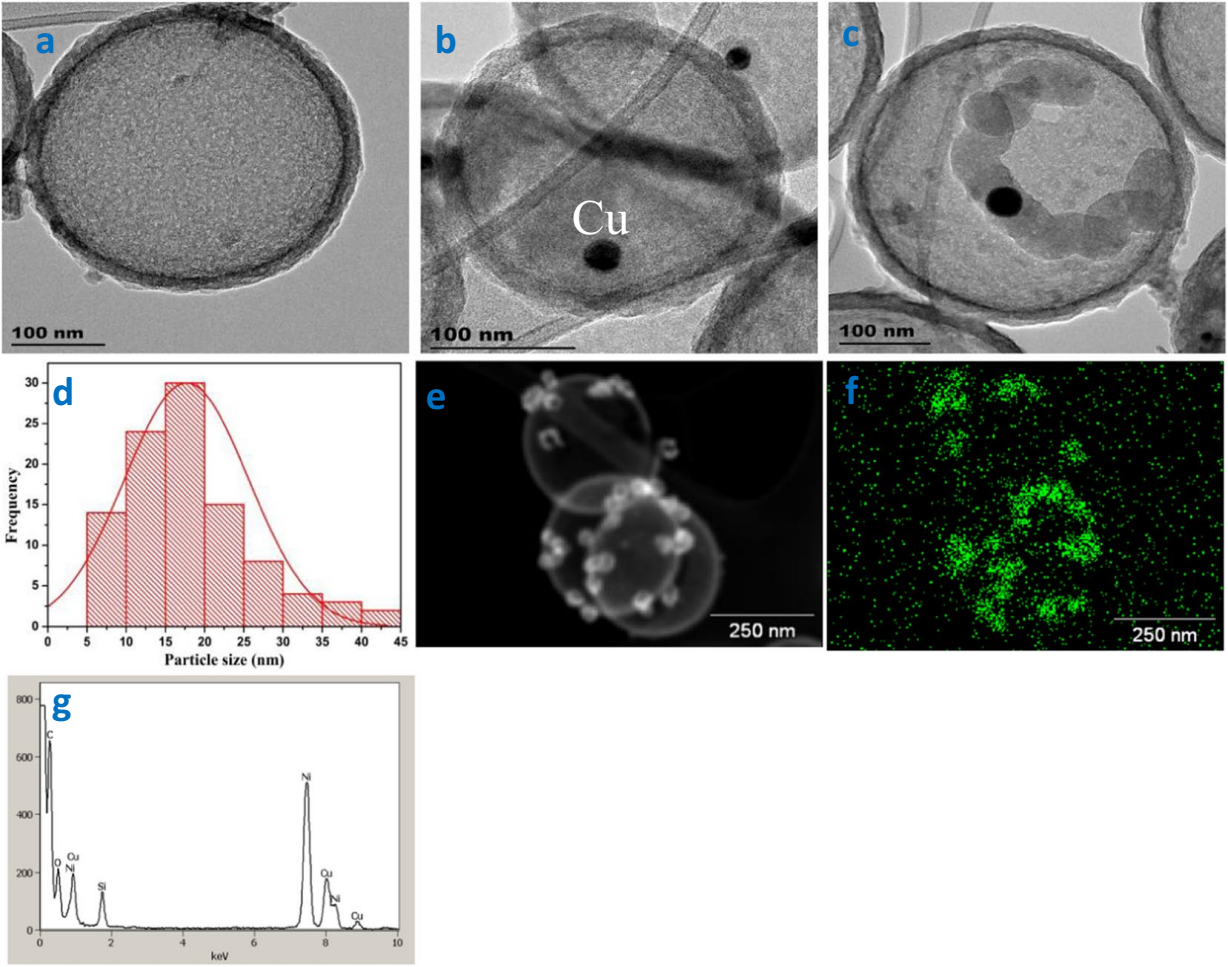
TEM images of (**a**) HCS, (**b**) Cu@HCS, (**c**) Cu@HCS and a carbon helix, growing from both sides of the Cu particle (black spot), (**d**) Cu particle size distribution in Cu@HCS. (1% Cu in HCSs; 300 °C, 30 mins; Cu reduction @ 400 °C). EDX data collected on Cu@HCS on a Ni grid showing (**e**) the dark field image (**f**) the Cu distribution in the HCSs and (**g**) the energy dispersive spectrum.

The HCSs were made by standard procedures and a range of techniques, including XRD (Supplementary Fig. S1), confirmed their structures. The techniques revealed the presence of carbon (and silica) in the HCS (and the HCS precursor). SEM images indicated that the materials were spherical with relatively uniform sizes and outer diameters of 300 ± 40 nm (silica) and 310 ± 40 nm (HCSs) for silica and HCSs respectively (Supplementary Fig. S2). Very few broken HCSs were observed (<3%). TEM images indicated that the HCSs had an inner diameter of 290 ± 30 nm (Fig. S2) revealing that silica shrinkage had occurred on thermal treatment during their synthesis, as reported by other workers (Figs 1a and 2a)^21^. The width of the carbon layer was measured to be 20 ± 5 nm. The surface analysis of the HCSs indicated that the material had a type IV isotherm with H1 hysteresis loop, according to the IUPAC classification, and was mesoporous (surface area of 568 m^2^g^−1^ and 3.7 nm pore size) (Supplementary Fig. S3, Supplementary Table S1).

Cu was added to the HCSs using a modified version of the procedure described in the literature^13^. The loading was performed using copper acetate (varying concentrations in water) in an autoclave at 100 °C for 24 h. Under the reaction conditions the Cu ions form CuO and some sintering occurs. In initial reactions, 5% and 10% Cu loadings (by mass) were used and Cu (as CuO) was deposited both inside and outside the HCSs. The number of particles contained in a HCS varied and the CuO particles tended to be large (30 ± 10 nm and 40 ± 10 nm for 5% and 10% loadings, respectively). Figure 1b shows Cu particles at 5% loading (arrows) both inside and outside a HCS. Reduction at 400 °C for 1 h resulted in conversion of the CuO to Cu and some sintering occurred. Figure 1c shows Cu particles (black dots) at 5% loading. The TEM images (Fig. 1c) also indicate no change is morphology of the HCS after Cu loading (d = 310 ± 40 nm).

In contrast, at a 1% Cu loading, almost all the Cu appears to reside inside the HCSs with formation of small CuO particles (see below). This sample was called CuO@HCS and was used in all further studies. The CuO particles (1% loading) were then reduced at 400 °C in H_2_ (flow rate; 100 mL min) for 1 h to produce Cu metal particles. This sample was called Cu@HCS. The Cu particles in the sample vary in size (5–45 nm, with an average size ca. 25 nm; Fig. 2d,e) and the number of particles in any HCS varied from zero to tens. An example of a HCS containing one Cu particle (14 nm) is shown in the TEM image in Fig. 2b. The presence of Cu was confirmed by EDX data (Fig. 2g and Supplementary Fig. S4). The CuO was reduced and then used to make the CNFs. The active catalyst is thus expected to be the Cu metal nanoparticles (Cu°), with information supported by temperature programmed reduction data (Supplementary Fig. S5) as well as results from previous studies on the growth of helical CNFs from Cu nanoparticles^17–19,22^.

BET analysis of the Cu@HCS material revealed that it had a type IV isotherm with H1 hysteresis loop and was mesoporous (3.3 nm pore size) (Supplementary Fig. S3). The changes in surface morphology between the HCS and Cu@HCS are assumed to be due to some pore blockage because of the loaded Cu particles. The Raman spectra for HCS and Cu@HCS are shown in Supplementary Fig. S6. The carbon material exhibited the expected two peaks at about 1330 cm^−1^ (D-band), and 1590 cm^−1^ (G-band)^23^. The relative intensity ratios (I_D_/I_G_) of the D-band and the G-band for HCS and Cu@HCS are 0.85 and 0.78 respectively, indicating that carbon materials are composed of small graphene sheets with some degree of graphitization and that the Cu could be acting as a catalyst to increase the graphitic nature of the carbon layer.

Not unexpectedly, due to the low concentration of Cu, the presence of the Cu by XRD analysis was not detected (Supplementary Fig. S1c). Similarly, XPS analysis did not detect Cu but this is also consistent with the Cu being placed inside the HCS where the electron beam cannot penetrate the carbon shell (wall thickness *ca*. 20 nm). The C (1 s) spectra could be deconvoluted into four peaks with binding energies of 288.7, 287.1, 285.0, and 284.2 eV (Supplementary Fig. S7, Supplementary Table S2). These correspond to C (1 s) of pure graphitic sites (284.2 eV), C-C sp3-hybridized carbon (285.0 eV), sp2-hybridized carbon in C=O (287.1 eV) and to O=C-O (288.7) (Supplementary Table S1).

Acetylene was then passed over the Cu@HCS for 30 mins (100 mL min^−1^) using different synthesis parameters at 300 °C, 325 °C and 350 °C and from these studies 300 °C was chosen as the temperature for further study as this produced less CNFs in the HCSs.

**Figure 3:**
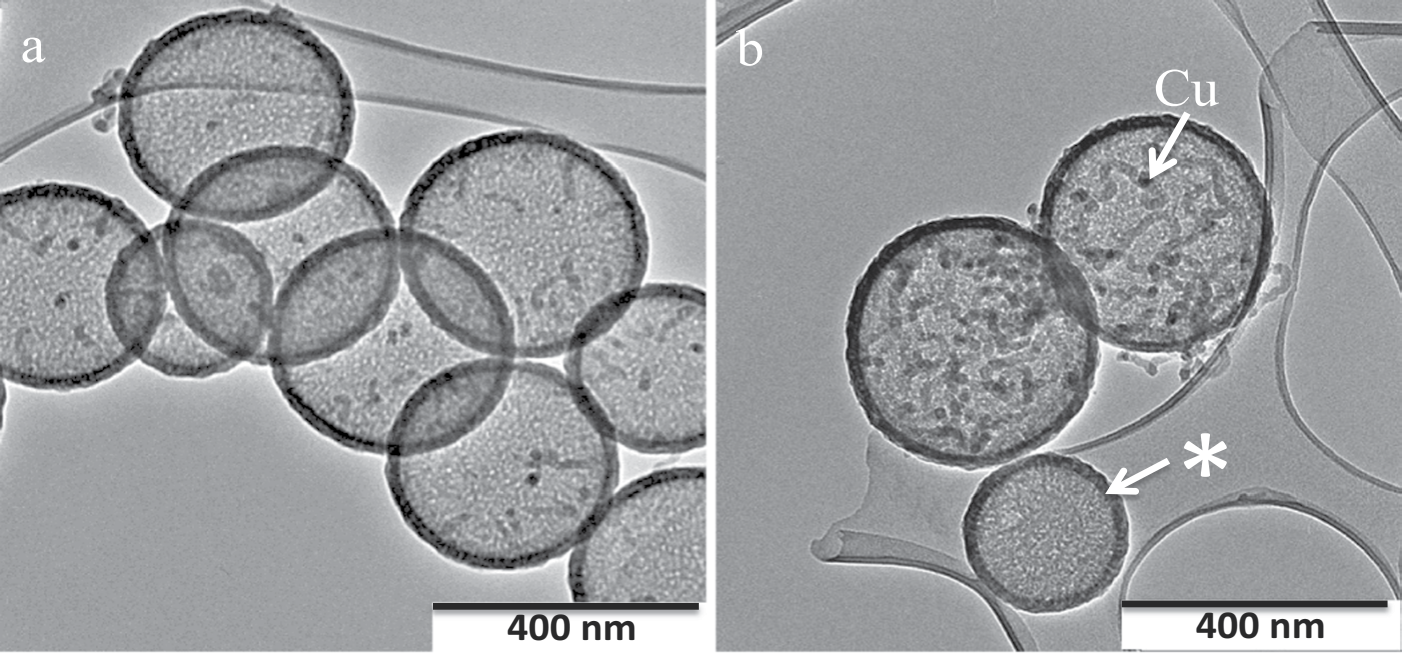
CNFs grown in HCSs (**a**) small Cu particles and short CNFs, (**b**) small Cu particles and longer CNFs (1% Cu loading, T = 300 °C, C_2_H_2_ flow rate 100 mL min for 30 mins). An HCS that contains no Cu particles is shown by *. Some CNFs can be seen growing outside the HCSs; due to Cu particles deposited on the outside of the carbon shell (**b**).

The growth process was studied in detail by TEM. A series of TEM images on CNFs produced at 300 °C, indicating the effect of Cu particle size on the CNF morphology, is shown in Fig. 3. The growth of the CNFs is seen to vary; in Fig. 3a only short CNFs are seen while in 3b longer, worm like CNFs are evident. The lengths and sizes are associated with the sizes of the Cu particles found in the HCSs (see below) as well as the reaction time. From the images shown in Fig. 3 it can be seen that (i) when HCSs contain small Cu particles (ca. 14 nm) the fibres are short, growing only from one side of the Cu and are worm like rather than helix shaped (Fig. 3a), (ii) CNFs that grow from larger Cu particles still do not show helix formation but are more coiled (Fig. 3b), (iii) this growth is more clearly seen in Supplementary Fig. S8 where a single Cu particle is seen to grow a single CNF from one side of the particle and (iv) the HCS constrains the growth of the CNF to fit the HCS (Supplementary Fig. S9).

**Figure 4:**
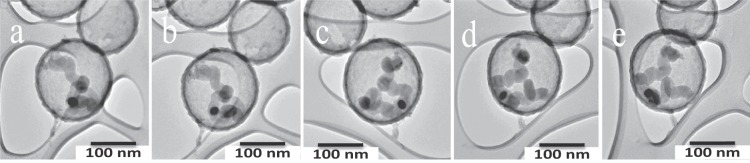
TEM tilting (alpha) experiments showing helix movement in a HCS (**a**) −58° (**b**) −30° (**c**) 0° (**d**) +30° (**e**) +58 °C.

It was also observed that larger Cu particles (25 nm) grow carbon helices and the helices grow from the two sides of the Cu particle (Fig. 2c). Here symmetrical carbon helices can be seen growing from two sides of a Cu particle. This is the growth expected for helices from large Cu particles and indicates that the growth mechanism for the helices inside the HCS is similar to that for growth outside the HCS^15,16^. Further examples of helix growth and the effect of the HCS constraint on this growth are seen in Fig. 4 and Supplementary Fig. S10.

Variation of synthesis conditions to make the CNFs as well as the variation of the Cu particle sizes thus produced a wide range of well-separated CNF structures for analysis. The Cu particle size, CNF diameter, coil pitch (Supplementary Fig. S11) and where possible, the fibre length of over 200 CNFs were thus measured and tabulated and the averaged data are shown in Table 1. While the fibres appear to be contained within the HCS (Figs 2c and 3b; and seen in many other images obtained), this was further confirmed by TEM tilting experiments. The TEM images were tilted around both the alpha (58°) and beta directions (28°) and a series of images for the alpha tilting experiments are shown in Fig. 4. The single Cu particle and the carbon helix are clearly seen to reside in the HCS and the data are consistent with containment in the HCS.

**Table 1:** Carbon fibre helices dimensions obtained from different studies.

Catalyst	Reaction conditions	Catalyst Size (nm)	Helix diam. (nm)	Coil pitch (nm)	Growth shape	Ref
Cu/TiO_2_	H_2_/C_2_H_2_ 250 °C, 1 h.	50–100 100–500	Various shapes	—	Helical linear	^19^
CuO or CuO/SrO	H_2_/C_2_H_2_ or N_2_/C_2_H_2_ 300 °C for 30 min	—	50–90	—	helical	^20^
Copper nitrate unsupported	H_2_/C_2_H_2_ 250 °C for 1 h	55	50	115	helical	^22^
Cu tartrate, butyrate, oxalate, and lactate; unsupported	H_2_/C_2_H_2_ 250 °C for 30 min	30–80	100	1000	Irregular shape	^24^
Cupric tartrate unsupported	C_2_H_2_ 280°–480 °C for 40 min	—	100–200	500–1000	From linear to helical	^25^
Co on SiO_2_ by atomic layer deposition	C_2_H_2_; 260 °C for 20 min	5–35 35–50 50–80 > 80	10–40 40–60 60–120 90–250		linear linear/helical helical linear	^26^
Cu-Ni alloy nanoparticles catalysts	C_2_H_2_ 241 °C	40	100	—	helical	^27^
Copper tartrate and CuO	H_2_/C_2_H_2_ 200–250 °C for 3–30 min	30–60 60–120	—	—	helical linear	^28^
Cu_2_O nano particles	C_2_H_2_ 265 °C for 30 min	50	400–500		Linear (10–20 microns)	^29^
Copper tartrate	H_2_/C_2_H_2_ 400 °C for 24 min	—	100–400	—	Coiled but not helical	^30^
Copper acetate, HCS support	H_2_/C_2_H_2_ 300–350 °C; 5 to 60 min	<10 10–20 20–40	<12 < 25 < 45	na na 40–70	linear linear/twist helix	Present study

Our data are similar to earlier literature reports which suggested that helical coils only grew when the Cu particle size exceeded a certain minimum size, about d = 35 nm^20,24,25^. In our case, the ability to control the sintering of Cu has allowed for helix formation to occur from even smaller Cu particles (25 nm and less; see Table 1), but with poorly defined helices. The data are consistent with literature values for helices grown from Cu outside a HCS. Because of the ease of visualising the Cu and helices within a HCS, the ability to correlate growth with Cu particle size is facile.

From a consideration of an extensive number of TEM images that were measured in this study, some conclusions can be drawn about the growth of carbon helices inside a HCS.

**Figure 5:**
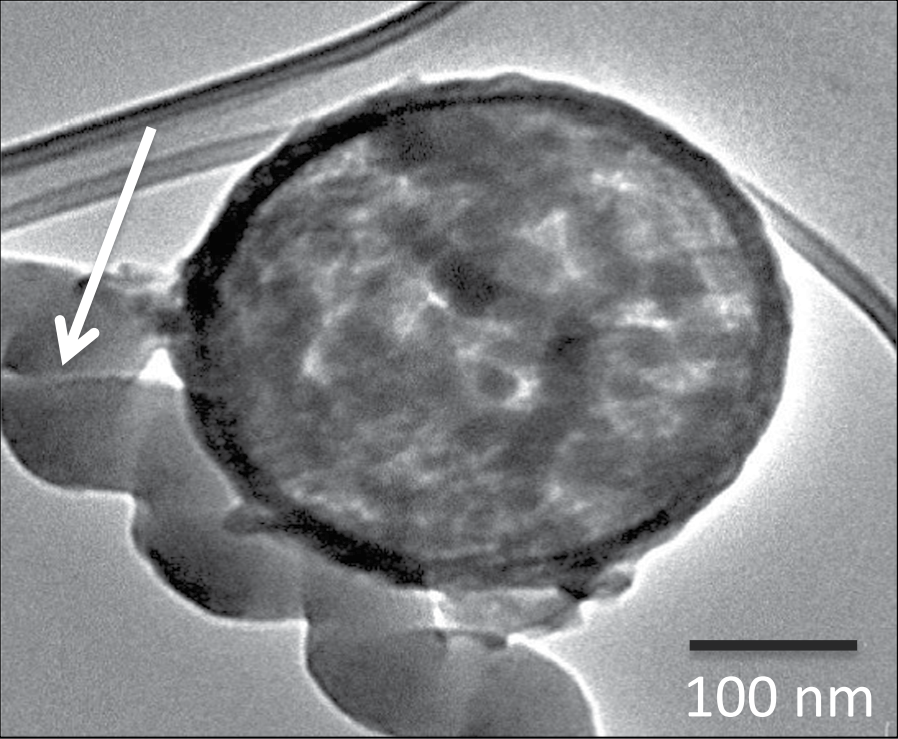
HCS completely filled with CNFs. (Note the large linear carbon fibre, shown by an arrow, formed by a Cu particle outside the HCS (d_HCS_ = ca. 300 nm; d_coil outside the CNF_ = 50 nm; growth conditions: T = 300 °C; 1% Cu loading).
viii) Eventually the HCS can be completely filled by the helix or helices (Fig. 5). The coiled CNF with d = 20 nm has filled the interior of the HCS. It will be interesting to see if the HCS can be destroyed by the helices when growth produces more carbon than can be accommodated within the HCS.

i) Small Cu particles (<10 nm) lead to ‘worm like’ CNFs (see Fig. 3a) with the Cu particle growing from one side of a Cu particle with coil lengths <100 nm
ii) Cu particles between 10–20 nm in diameter indicate CNF growth from one side of the Cu particle but with a twisted shape (non-linear but with little curvature) and lengths between 50–150 nm. (Fig. 3b; Supplementary Fig. S7)
iii) Cu particles >ca. 25 nm show clear CNF helix formation but with a large coil pitch (40–70 nm) (see Fig. 2c, Supplementary Fig. S9)
iv) No particles with size >50 nm were observed under our synthesis conditions – but the CNFs formed here would be expected to have a classical helix structure (see Table 1 and references therein).
v) The Cu particle size is always smaller that the CNF size i.e. d_Cu_ < d_CNF_. (This can be readily be seen in Figs 2c and 4)
vi) Attempts to correlate the Cu particle shape of the small Cu particles with the change in CNF morphology, have to date, not been successful^19^.
vii) A key feature to note is that whereas the helix generated outside a HCS grows linearly, inside the HCS the carbon boundary layer restricts this growth leading to CNFs that are not linear. The carbon HCS boundary layer forces the CNFs to bend. This is seen in Fig. 5 where a well formed ‘linear’ helical CNF is seen growing outside a HCS that contains smaller helices.

While this paper provides a specific example for the growth of materials inside a HCS the general implications from the study relate to the following: (i) the possibility of building homogeneous catalysts that are contained within a HCS that can be filtered after use, (ii) retention of small heterogeneous materials within a HCS that would normally be difficult to filter, and (iii) limited sintering of metals within a HCS to provide easy control of the size of a catalyst particle.

In this study, we have shown that carbon fibre helices materials can be constructed within a HCS. In other preliminary studies we have also been able to grow other carbon shaped materials inside a HCS e.g. CNTs (Fe/Co catalyst) and regular CNFs (Ni catalyst).

## Methods

### Starting Materials

All the chemicals used were of analytical grade. Tetraethylorthosilicate (TEOS), copper acetate, resorcinol, formaldehyde were all procured from Sigma Aldrich. Ammonia, ethanol, hydrofluoric acid and were obtained from Merck Chemicals.

### Synthesis of hollow carbon spheres (HCSs)

Hollow carbon spheres were synthesized by a hydrothermal method. In a typical reaction, 2.5 ml of TEOS was added into a solution of 60 ml of ethanol, 10 ml of water and 2.5 ml of ammonia. The mixture was stirred for 30 min at room temperature. After that 0.4 g of a resorcinol and 0.2 g of formaldehyde mixture were then added. The solution was stirred at room temperature for 24 h and transferred to an autoclave. The product from the autoclave was then dried in an oven at 100 °C for 24 h. The obtained product was centrifuged, washed with water and ethanol, and then dried at 60 °C overnight. The obtained yellow sample was carbonized at 700 °C under nitrogen flow for 6 h with heating rate of 5 °C min^−1^. The carbon-silica composite obtained after pyrolysis was etched with hydrofluoric (HF) acid at room temperature, in order to remove the silica template. The template-free hollow carbon sphere product thus obtained was then filtered, washed with ethanol and dried at 60 °C.

### Preparation of Cu@HCS

The Cu@HCS catalyst was prepared by hydrothermal impregnation. Typically, 0.1 g HCS and 0.031 g of copper acetate were homogenously dispersed in 30 ml water and ultrasonicated for 30 min. The mixture was transferred into an autoclave and heated at 100 °C for 6 h. The product was then washed with water and ethanol, and dried in an oven overnight at 60 °C to give CuO@HCS. The obtained product was reduced with hydrogen (100 ml min^−1^) at 400 °C for 2 h in a tube furnace. The product was denoted as Cu@HCS.

### Synthesis of carbon helices

Acetylene was passed over the Cu@HCS catalysts at 300–300 °C for times ranging from 5 mins to 1 h. For example, the catalyst was first activated by heating at 10 °C min^−1^ under hydrogen (100 ml min^−1^) until a temperature of 300 °C was reached. Then acetylene (100 ml min^−1^) was passed over the substrate for 1 h. The substrate was then cooled to room temperature under hydrogen and then characterised.

### Catalyst characterization

XRD measurements were carried out on a Brucker D2 phaser powder X-ray diffractometer with a Ni-filtered Cu Kα radiation source (λ = 1.5406 Å) operating at 30 kV and 10 mA, with a scanning rate of 3° min^−1^ at 2θ between 10°−90° respectively. The surface morphology of the prepared material was studied using a Philips XL-30 SEM instrument. Transmission electron microscopy (TEM) analysis of the carbon materials was performed on either a JEOL JEM 100 s, or FEI Tecnai G^2^ spirit electron microscope operated at 200 KeV with a point-to-point resolution of 2.3 Å. To establish whether the carbon fiber did indeed grow inside the hollow sphere, tilting experiments were carried out using the FEI Tecnai G2 Spirit transmission electron microscope at 120 kV using a zero background beryllium double tilt holder. Samples were tilted around both alpha and beta axes, taking images at different tilt angles with maximum tilt of the alpha axis at 58 degrees and that of the beta axis at 60 degrees. Raman spectra was recorded using a SENTERRA system attached with a He–Ne laser (532 nm) as the excitation source having an output power of 15 mW with a laser beam spot size of 100 µm using an appropriate lens system. Nitrogen adsorption-desorption isotherm measurements were performed at 77 K using a Micrometrics Tritan 3000 surface area analyzer. Prior to adsorption, the samples were evacuated at 523 K for 12 h. The specific surface area of the samples was estimated using the Brunauer-Emmett-Teller (BET) method and the pore size was calculated by Barrett-Joyner-Halenda (BJH) formula. The pore volume data were determined from the amount of nitrogen adsorbed at P/Po = 0.5.

## Supplementary information

09b supp material
