# Supplementary material for: Building carbon structures inside hollow carbon spheres
Source: Sci Rep. 2019 Jul 23;9:10642. doi: 10.1038/s41598-019-46992-1 (PMC6650401; doi:10.1038/s41598-019-46992-1)
Supplement: Supplementary file 1 — 09b supp material [file 41598_2019_46992_MOESM1_ESM.pdf]

Supplementary information

Building carbon structures inside hollow carbon spheres

Prakash M. Gangatharan,[a] Manoko S Maubane [a,b] and Neil J Coville [a]*

[a] DST-NRF Centre of Excellence in Strong Materials and the Molecular Sciences Institute, School of Chemistry, University of the Witwatersrand, 2050, Johannesburg, South Africa
[b] Microscopy and Microanalysis Unit, University of the Witwatersrand, 2050, Johannesburg, South Africa (email: neil.coville@wits.ac.za)

Experimental procedure for the tilting experiments

To establish whether the carbon fiber did indeed grow inside the hollow sphere, tilting experiments were carried out using a FEI Tecnai G2 Spirit transmission electron microscope at 120 kV. In a standard experiment, about 2 mg of a sample to be analyzed was placed in a bullet Eppendorf tube containing 10 mL of methanol. The mixture was then sonicated for 10 minutes to give a homogeneous suspension of CNTs in the solvent. A drop of the suspension was then spread on a carbon copper grid (200 mesh) and allowed to dry at room temperature. The grid was then mounted onto a zero background beryllium double tilt holder which was placed into the TEM chamber, ready for viewing. Samples were tilted around both alpha and beta axes, taking images at different tilt angles. In our experiments, the maximum tilt of the alpha axis was 58 degrees and that of the beta axis was 28 degrees.

List of supplementary tables

Table S1. Catalyst textural properties
Table S2. XPS data for carbon

List of supplementary figures

Figure S1. XRD patterns of (a) SiO_2, (b) HCSs and (c) Cu@HCS (1% Cu loading)
Figure S2. SEM images of (a) SiO_2, (b) HCSs and (c) Cu@HCS (1% Cu loading)
Figure S3. BET data for HCSs
Figure S4. Map of element distribution of Cu@HCS on a Ni grid showing the (a) dark field image, and elements (b) C, (c) Ni, (d) O, (e) Si, (f) Cu and (g) a composite of the elements. The energy dispersive spectrum is shown in (h).
Figure S5. Temperature programmed reduction profile for 5%CuO@HCS. The data indicate the conversion of CuO to Cu occurs at ca 195 ^oC. The negative peak at T ca 700 ^oC is due to the methantion of the HCS. (Data were obtained on a Micromertics Auto Chem II unit under 5 % H_2/Ar at a rate of 10 °C.min^{-1} from 50 to 850 °C. The gas flow rate of 50 mL.min^{-1} through the reactor was controlled by three Brooks mass flow controllers)
Figure S6. Raman spectra of (a) HCSs and (b) Cu@HCS (1% Cu loading)
Figure S7. XPS spectra of (a) Cu@HCS (1% Cu) full scan and (b) C1s scan

Figure S8. (a) CNF grown from one side of a single Cu particle (see arrow). (b) Four CNFs grown in a single HCS, also with growth from one side of the Cu particle. The CNFs show curvature (c) Growth from eight CNFs (1%Cu/HCS; 300 ^{o}C, 30 mins)
Figure S9. CNF growth from Cu showing that the CNF growth directions are influenced by the HCS size and shape. (1%Cu/HCS; 300 ^{o}C, 30 mins)
Figure S10. CNF helix growth inside a HCS from a single Cu particle. (a) The CNF shows a helix close to the Cu particle but a linear structure further away. This is expected and relates to the dynamic change in shape of the Cu particle with time (see ref 19), (b) a CNF showing some coiling as well as linear growth regions that show CNF bending due to the HCS shape/size. (1%Cu/HCS; 300 ^{o}C, 30 mins) .
Figure S11. Definition of coil dimensions. Taken from: Shaikjee A. & Coville, N.J. The synthesis, properties and uses of carbon materials with helical morphology. *J. Adv. Res.* **3,** 195–223 (2012)

Supplementary Tables

Table S1. Catalyst textural properties

catalyst	S_{BET}[a] m^2/g	Pore volume cm^3/g	Pore size nm
HCS	568	1.10	3.7
Cu@HCS (1% Cu)	526	0.92	3.3

[a] S_{BET} is calculated by the Brunauer-Emmet-Teller (BET) method

Table S2. XPS data for carbon

Element	Binding Energy (eV)	Core level	% atom
C1s	288.7	O=C-O	1.5
C1s	287.1	C=O	1.5
C1s	285.0	C-C	88.7
C1s	284.2	C=C	8.3

Supplementary Figures

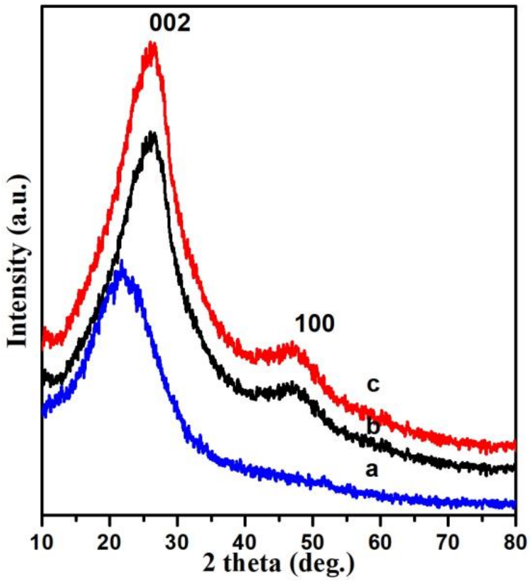

Figure S1. XRD patterns of (a) SiO_2, (b) HCSs and (c) Cu@HCS (1% Cu loading)

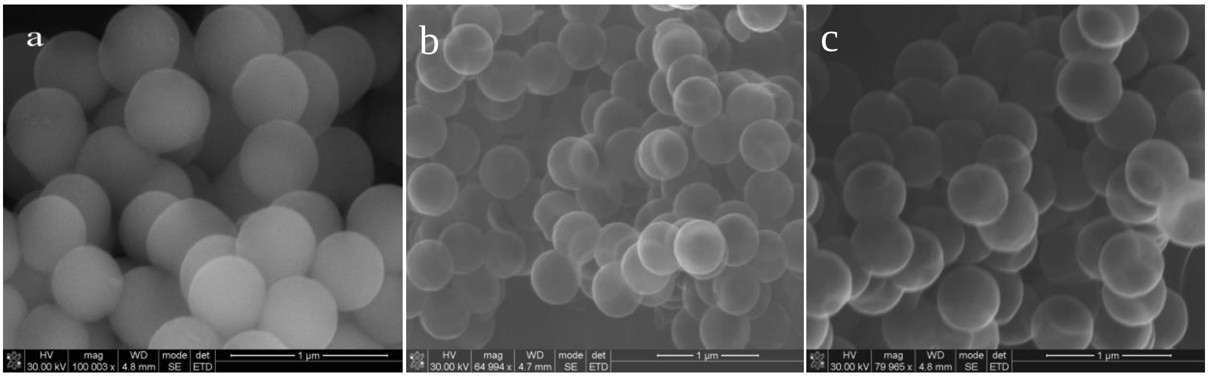

Figure S2. SEM images of (a) SiO_2, (b) HCSs and (c) Cu@HCS (1% Cu loading)

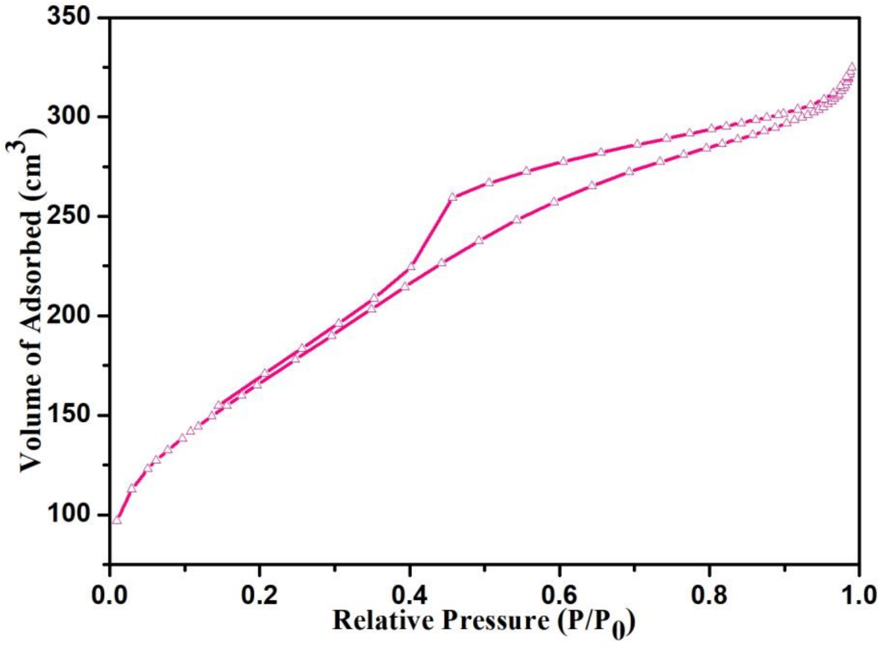

Figure S3. BET data for HCSs

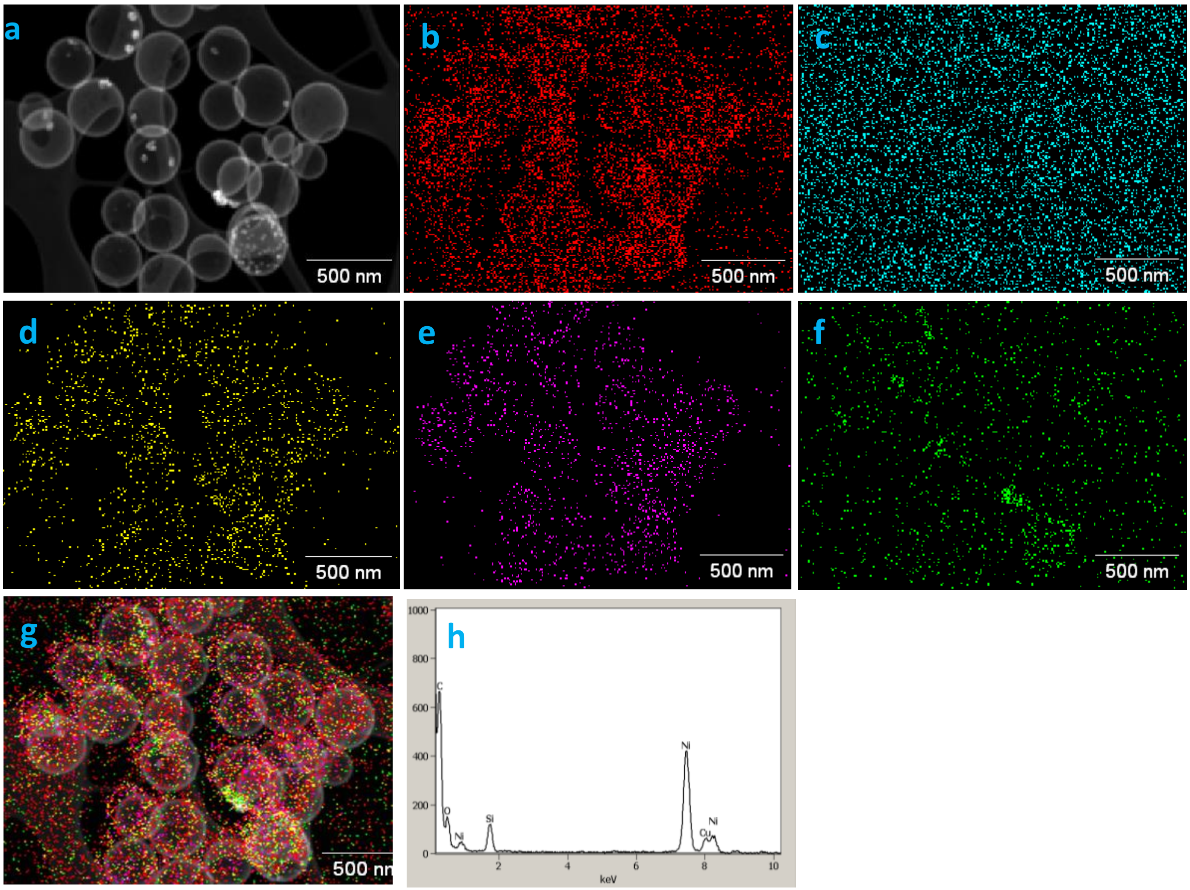

Figure S4. Map of element distribution of Cu@HCS on a Ni grid showing the (a) dark field image, and elements (b) C, (c) Ni, (d) O, (e) Si, (f) Cu and (g) a composite of the elements. The energy dispersive spectrum is shown in (h).

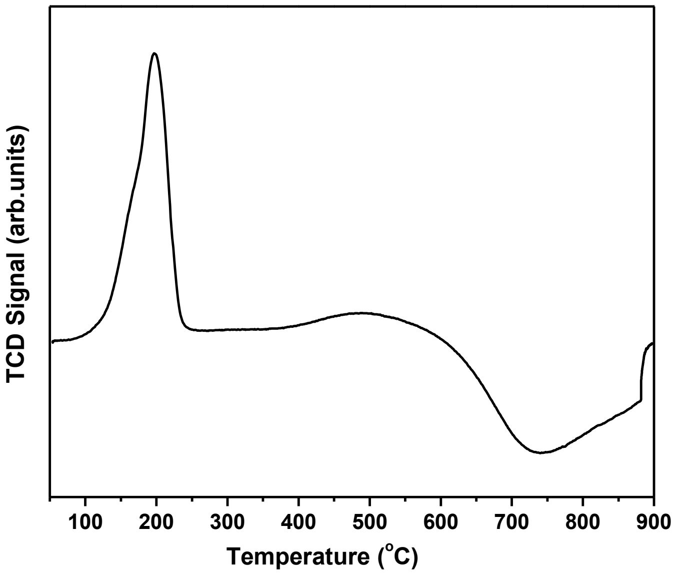

Figure S5. Temperature programmed reduction profile for 5%CuO@HCS. The data indicate the conversion of CuO to Cu occurs at ca 195 °C. The negative peak at T ca 700 °C is due to the methantion of the HCS. (Data were obtained on a Micromertics Auto Chem II unit under 5 % H_2/Ar at a rate of 10 °C.min^{-1} from 50 to 850 °C. The gas flow rate of 50 mL.min^{-1} through the reactor was controlled by three Brooks mass flow controllers)

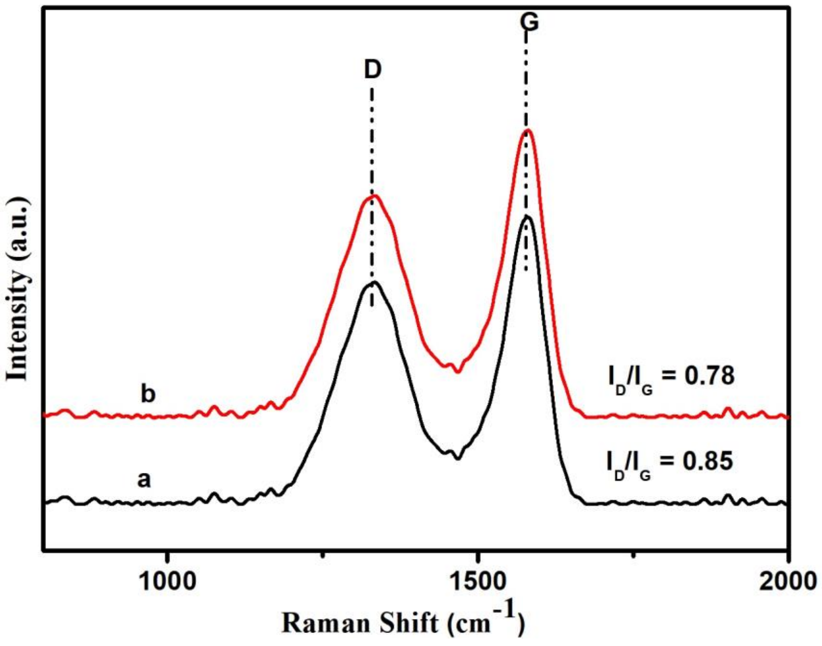

Figure S6 Raman spectra of (a) HCSs and (b) Cu@HCS (1% Cu loading)

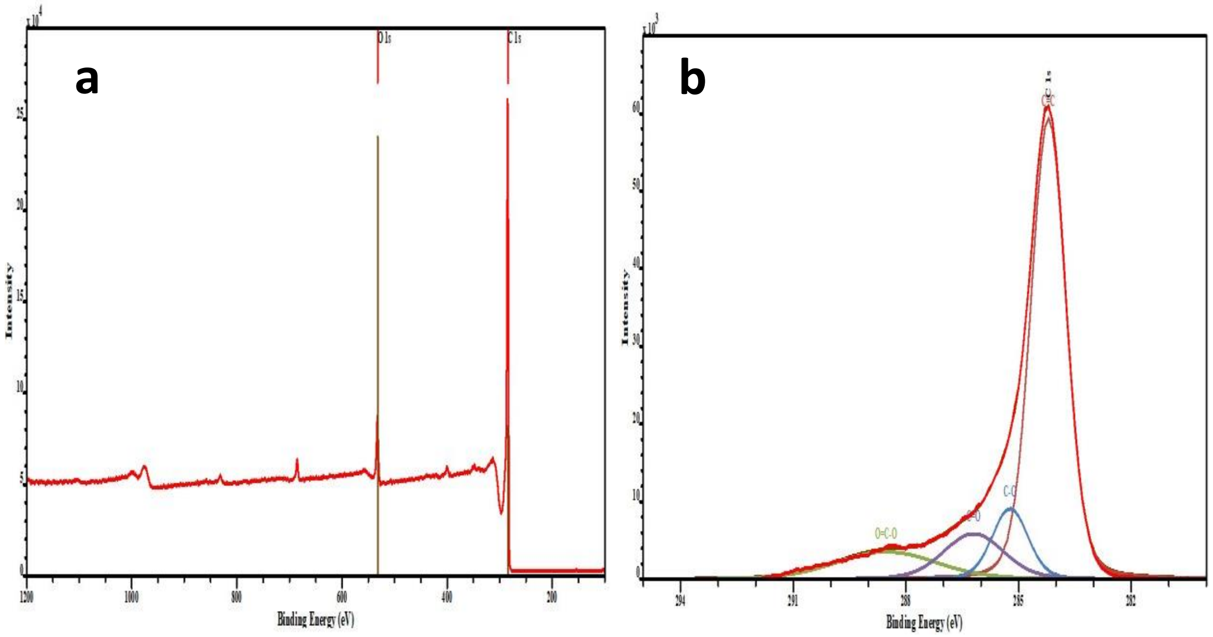

Figure S7. XPS spectra of (a) Cu@HCS (1% Cu) full scan and (b) C1s scan

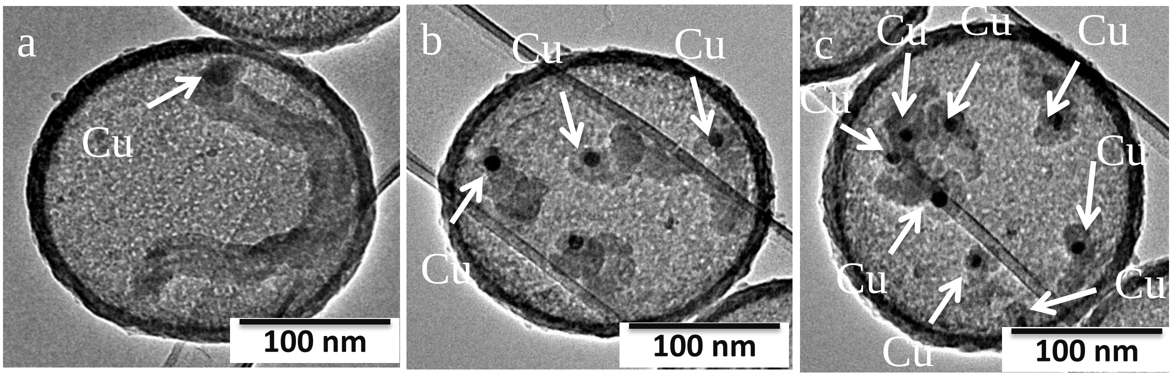

Figure S8. (a) CNF grown from one side of a single Cu particle (see arrow). (b) Four CNFs grown in a single HCS, also with growth from one side of the Cu particle. The CNFs show curvature (c) Growth from eight CNFs (1%Cu/HCS; 300 ^{o}C, 30 mins)

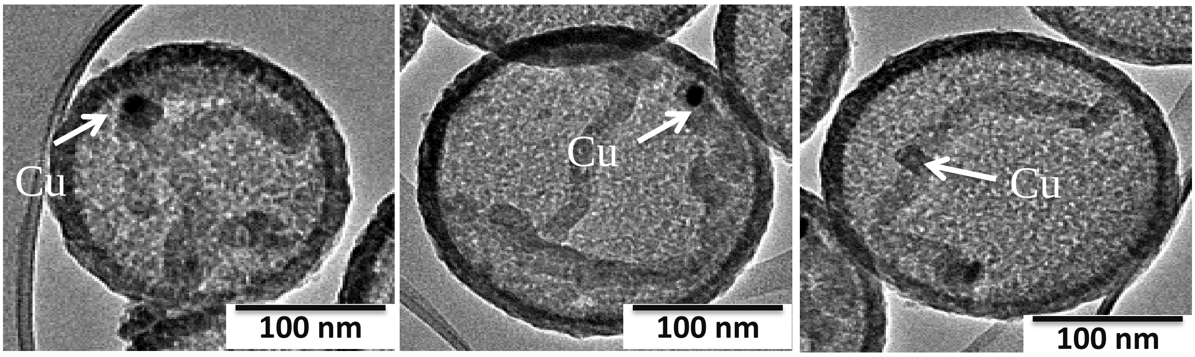

Figure S9. CNF growth from Cu showing that the CNF growth directions are influenced by the HCS size and shape. (1%Cu/HCS; 300 ^{o}C, 30 mins)

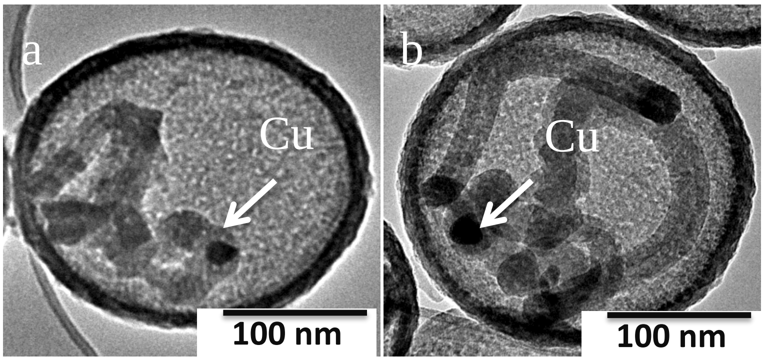

Figure S10. CNF helix growth inside a HCS from a single Cu particle. (a) The CNF shows a helix close to the Cu particle but a linear structure further away. This is expected and relates to the dynamic change in shape of the Cu particle with time (see ref 19), (b) a CNF showing some coiling as well as linear growth regions that show CNF bending due to the HCS shape/size. (1%Cu/HCS; 300 ^{o}C, 30 mins)

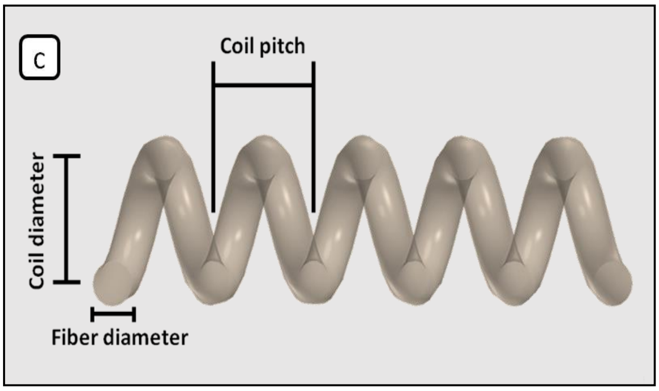

Figure S11. Definition of coil dimensions. Taken from: Shaikjee A. & Coville, N.J. The synthesis, properties and uses of carbon materials with helical morphology. *J. Adv. Res.* **3,** 195–223 (2012)
